# The kynurenine pathway in schizophrenia, bipolar disorder, and major depressive disorder: a systematic review and meta-analysis of cerebrospinal fluid studies

**DOI:** 10.1192/j.eurpsy.2023.1291

**Published:** 2023-07-19

**Authors:** E. Salagre, B. S. Fernandes, M. E. Inam, I. Grande, E. Vieta, J. Quevedo, Z. Zhao

**Affiliations:** 1 Department of Affective Disorders, Aarhus University Hospital, Aarhus, Denmark; 2 Center for Precision Health, School of Biomedical Informatics, The University of Texas Health Science Center at Houston, Houston, United States; 3 Bipolar and Depressive Disorders Unit, Hospital Clinic, Institute of Neurosciences, University of Barcelona, IDIBAPS, CIBERSAM, Barcelona, Spain; 4 Faillace Department of Psychiatry and Behavioral Sciences, McGovern Medical School, The University of Texas Health Science Center at Houston, Houston, United States

## Abstract

**Introduction:**

The kynurenine pathway has been suggested to be involved in the pathophysiology of psychiatric disorders, including schizophrenia (SZ), bipolar disorder (BD), and major depressive disorder (MDD).

**Objectives:**

To conduct a systematic review and meta-analysis of the kynurenine pathway metabolites from cerebrospinal fluid samples in SZ, BD, and MDD.

**Methods:**

PubMed and Scopus databases were searched from inception to April 2022 to identify case-control studies assessing kynurenine metabolites [tryptophan (TRP), kynurenine (KYN), kynurenic acid (KA), quinolinic acid (QA), and 3-hydroxykynurenine (3-HK)] in SZ, BD, and MDD vs. healthy controls (HC). PRISMA guidelines were followed in the literature review. The random effects model parameter was selected when comparing the standardized mean differences (SMD) between groups.

**Results:**

A total of 23 articles met inclusion criteria (number of articles k=8, 8, 11 for SZ, BD, and MDD, respectively). For SZ, KA levels were increased in SZ compared to HC (SMD=2.64, CI=1.16-4.13, p=0.0005, I^2^=96%, k=6, n=384). KYN levels were not significantly different between SZ and HC (SMD=4.19, CI=-0.70 to 9.09, p=0.0933, I^2^=99%, k=4, n=188). For BD, TRP levels (k=7) did not differ significantly between BD and HC. There was a limited number of studies to conclude increased levels of KA in BD (k=2). For MDD, although some studies tended toward increased levels of KYN in those with remission vs. decreased levels in those with current depression, there were no significant differences in any of the kynurenine metabolite levels. There was a limited number of studies to conclude increased levels of QA in MDD (k=2).

**Conclusions:**

KA, which has possibly neuroprotective effects, is increased in SZ. QA, which has neurotoxic effects, may be increased in MDD. There may be alterations in this pathway based on population characteristics and mood states.

**Disclosure of Interest:**

E. Salagre: None Declared, B. Fernandes: None Declared, M. Inam: None Declared, I. Grande Grant / Research support from: Spanish Ministry of Science and Innovation (MCIN) (PI19/00954) integrated into the Plan Nacional de I+D+I and cofinanced by the ISCIII-Subdireccion General de Evaluacion y el Fondos Europeos de la Unión Europea (FEDER, FSE, Next Generation EU/Plan de Recuperación Transformación y Resiliencia_PRTR); the Instituto de Salud Carlos III; the CIBER of Mental Health (CIBERSAM); and the Secretaria d’Universitats i Recerca del Departament d’Economia i Coneixement (2017 SGR 1365), CERCA Programme / Generalitat de Catalunya as well as the Fundació Clínic per la Recerca Biomèdica (Pons Bartran 2022-FRCB_PB1_2022), Paid Instructor of: ADAMED, Angelini, Casen Recordati, Ferrer, Janssen Cilag, and Lundbeck, Lundbeck-Otsuka, Luye, SEI Healthcare , E. Vieta Grant / Research support from: Spanish Ministry of Science and Innovation (PI15/00283, PI18/00805) integrated into the Plan Nacional de I+D+I and co-financed by the ISCIII Subdirección General de Evaluación and the Fondo Europeo de Desarrollo Regional (FEDER); the Instituto de Salud Carlos III; the CIBER of Mental Health (CIBERSAM); the Secretaria d’Universitats i Recerca del Departament d’Economia i Coneixement (2017 SGR 1365), the CERCA Programme, and the Departament de Salut de la Generalitat de Catalunya for the PERIS grant SLT006/17/00357, Paid Instructor of: AB-Biotics, AbbVie, Angelini, Biogen, Boehringer-Ingelheim, Celon Pharma, Dainippon Sumitomo Pharma, Ferrer, Gedeon Richter, GH Research, Glaxo-Smith Kline, Janssen, Lundbeck, Novartis, Orion Corporation, Organon, Otsuka, Sage, Sanofi-Aventis, Sunovion, and Takeda, J. Quevedo: None Declared, Z. Zhao: None Declared

